# Folie à deux, about a case

**DOI:** 10.1192/j.eurpsy.2022.1990

**Published:** 2022-09-01

**Authors:** M. Palomo Monge, M.F. Tascón Guerra, M. Pérez Fominaya, M.V. López Rodrigo, A. Osca Oliver, C. García Montero, V. Ros Font

**Affiliations:** 1 Hospital Nuestra Señora del Prado, Psiquiatria, Talavera de la Reina, Spain; 2 Hospital Nuestra Señora del Prado, Psiquiatría, Talavera de la Reina, Spain; 3 Hospital Provincial de Ávila, Servicio De Psiquiatría, Ávila, Spain

**Keywords:** folie à deux, schizophrénia, psychotic, shared psychotic disorder

## Abstract

**Introduction:**

First described in France by Lasègue and Falret as the presence of the same psychiatric symptom in 2 individuals. It involves the transference of delusional ideas from a “primary” affected individual to one or more “secondaries,” in close association.

**Objectives:**

We present the case of a patient, diagnosed with schizophrenia, who, after several years of evolution, and after a relapse, comes accompanied by his mother, who recently began to present the same delusional symptoms that the patient reported previously.

**Methods:**

After several pharmacological adjustments, control of the patient’s symptoms is achieved and it is he himself who is able to identify the symptoms that his mother presents, allowing her to also attend and be treated.

**Results:**

Induced delusional disorder F.24

**Conclusions:**

Among the variants that the folie a deux encompasses (folie imposée, folie communiquée, folie simultenée, folie induite) in this case we are probably talking about a folie communiquée, given the resistance over time of the patient’s mother until the symptoms develop. It is important to know these syndromes to be able to make a clear diagnosis, and depending on the way of onset and evolution, to be able to distinguish between the different subtypes, in order to avoid future complications and guide the corresponding treatment.

**Disclosure:**

No significant relationships.

